# [Expression of Concern] Increased expression of heat shock protein 90 under chemical hypoxic conditions protects cardiomyocytes against injury induced by serum and glucose deprivation

**DOI:** 10.3892/ijmm.2026.5821

**Published:** 2026-04-01

**Authors:** Keng Wu, Wenming Xu, Qiong You, Runmin Guo, Jianqiang Feng, Changran Zhang, Wen Wu

Int J Mol Med 30: 1138-1144, 2012; DOI: 10.3892/ijmm.2012.1099

Following the publication of the above paper, it was drawn to the Editor's attention by a concerned reader that the HSP90 blots shown in Figs. 2A and 4A on p. 1140 were strikingly similar after horizontally flipping, and then horizontally and vertically resizing, the bands in Fig. 2A, suggesting that these data panels were derived from the same original source where different experimental groups were reported.

The authors have been contacted by the Editorial Office to offer an explanation for this apparent anomaly in the presentation of the data in their paper, and we are awaiting their response. Due to the fact that we have been made aware of potential issues surrounding the scientific integrity of this paper, we are issuing an Expression of Concern to notify readers of this potential problem while the Editorial Office continues to investigate this matter further.

